# Exercise Training for Multiple Sclerosis: A Narrative Review of History, Benefits, Safety, Guidelines, and Promotion

**DOI:** 10.3390/ijerph182413245

**Published:** 2021-12-16

**Authors:** Yvonne Charlotte Learmonth, Robert Wayne Motl

**Affiliations:** 1 Discipline of Exercise Science, Murdoch University, Murdoch, WA 6150, Australia; 2 Centre for Molecular Medicine and Innovative Therapeutics, Centre for Healthy Ageing, Health Futures Institute, Murdoch University, Murdoch, WA 6150, Australia; 3 Perron Institute for Neurological and Translational Science, Nedlands, WA 6009, Australia; 4 Department of Kinesiology and Nutrition, University of Illinois Chicago, Chicago, IL 60612, USA; robmotl@uic.edu

**Keywords:** multiple sclerosis, exercise, controversy, guidelines, health promotion

## Abstract

Background: There have been significant advances in the medical treatment and management of multiple sclerosis pathogenesis, relapse and disease progression over the past 30 years. There have been advancements in the symptomatic treatment of multiple sclerosis, including management of secondary multiple sclerosis expressions such as walking, cognitive dysfunction, fatigue and depression. Scientific evidence and expert opinion suggest that exercise may be the single most effective non-pharmacological symptomatic treatment for multiple sclerosis. This article presents the historical context of exercise training within the multidisciplinary management of multiple sclerosis. We guide neurologists and healthcare providers on the recommended prescription of exercise and practical, theoretical methods to overcome barriers to exercise. Method: We undertook a critical search of the historical and current literature regarding exercise and multiple sclerosis from the viewpoint of exercise promotion by neurologists and the multidisciplinary care team. Results: We highlight the ever-strengthening body of research indicating that exercise is safe and effective for improving symptoms of multiple sclerosis. Further, exercise training may be necessary for reducing disease progression. Conclusion: We seek to encourage neurologists and specialists in multidisciplinary healthcare teams to prescribe and promote exercise at diagnosis and across all stages of the disease trajectory using prescriptive guidelines as part of comprehensive MS care. Available tools include clinical education to dispel any historical myths related to exercise in multiple sclerosis, clinical exercise guidelines and behaviour change theory to overcome patients barriers to exercise.

## 1. Introduction

Working within a multidisciplinary care team, clinical neurologists are primarily responsible for the long-term care of multiple sclerosis (MS) patients [1]. MS is a disorder of the central nervous system which has grown in prevalence to now impact over 2.6 M individuals [2]. The hallmark of the disease and its progression is neural inflammation and destruction of myelin tissue, resulting in central nervous system scarring and inefficient neural activity [3]. The pathological processes of the disease compound with psychological and environmental factors unique and significant to each individual and result in a plethora of symptoms and lifestyle impacts. Fatigue, mobility disability, pain, altered mood (e.g., depression) and cognitive decline are common in persons with MS [2,3], and overall quality of life and social participation is reduced. It is due to the combination of these reasons that persons with MS may have lower levels of physical activity and exercise in comparison with other populations [4,5,6,7].

Healthcare reports from the United States indicated that upwards of 50% of people with MS visit with a neurologist annually for ongoing care, and 25% of people with MS further utilise physiotherapy and occupational therapy [8]. Such observations suggest that neurologists and other rehabilitation specialists are well-positioned for ongoing counselling on rehabilitation, particularly exercise training in MS. The multidisciplinary team has an essential role alongside the broader MS community in caring for people with MS. We believe that the neurologist can play a critical role in endorsing and promoting exercise training in MS, and that specialist members of the team (e.g., MS nurses, physiotherapists, occupational therapists, exercise physiologists and behaviour change specialists) are pivotal in actioning exercise promotion. The focus on exercise prescription could be a salient part of therapy in MS care, as it offers many symptomatic and disease management benefits.

We note that physical activity is a behaviour broadly defined as “any bodily movement produced by contraction of skeletal muscle that results in a substantial increase in energy expenditure,” [9] and exercise is a subtype of physical activity. Exercise is a behaviour defined as a “planned, structured, and repetitive form of physical activity with the intention or goal of maintaining or improving one’s fitness and/or health” [9]. This distinction is paramount for emphasising and contextualising the current knowledge and associated recommendations on exercise as a behaviour among people with MS.

The benefits of exercise in improving physical performance, mental function and general wellbeing are unequivocal; we now know these benefits extent to possible neuroprotection of the nervous system. Growing research in human studies indicates the neuroprotective role of exercise against stroke, Parkinson’s disease and MS [10,11]. In persons with MS, exercise can decrease neural apoptosis and neurodegeneration, and may be effective at stimulating neuroplasticity, as overall exercise increases neurological functioning [10]. In addition to neuroprotection against disease, exercise is beneficial in persons with neurodegenerative disease. Experimental animal models of MS indicate strong evidence of exercise-induced changes in neural structure and function; however, translation to human studies has yet to show this outright [12]. In human studies, evidence from exercise intervention studies indicates exercise improves outcomes measured by neural imaging and improves peripheral biomarkers associated with neural health [13], and that exercise may assist in overall brain preservation [14].

Persons with MS express more interest in wellness topics than pharmacological therapy for disease management [15]. They have identified healthcare providers, particularly neurologists, as essential sources of information on wellness behaviours, particularly exercise training [16,17,18,19,20,21]. Healthcare providers, particularly neurologists, have further acknowledged their own central role in promoting exercise training among MS patients [21,22,23]. The wider multidisciplinary team of neurology clinicians, including advanced practice providers such as physiotherapists, occupational therapists, and exercise physiologists, alongside specialist nursing staff and professionals trained in behaviour change (e.g., psychologists) are critical in initialising and facilitating exercise behaviour change among persons with MS [24]. The patient-provider interaction can become a bedrock component of promoting and advising on exercise participation in MS. Facilitating such promotion of healthcare providers’ awareness and consistent usage of MS-specific evidence-based exercise guidelines, which acknowledges the capacity of persons with MS, is a vital tool in the management of MS.

The role of the healthcare provider and the patient-provider interaction to promote exercise is of high importance, as there is a current public health concern surrounding low physical activity levels in people with MS [4,5,6,7]. This public health concern has been underlined with recent events, including the COVID-19 pandemic where evidence indicates exercise levels may have declined even more for persons with MS [25]. One possible reason for this may be that many healthcare providers are not clearly or consistently advising patients on the importance of exercise, as physical activity, in the management of MS. We reach this conclusion from two primary viewpoints. The rate of physical activity in people with MS has not changed in over 30 years [5,26], despite expanding evidence of benefits for this behaviour. People with MS, who engage with healthcare providers more regularly than the general population, participate in substantially less physical activity than healthy controls from the general population [5,6].

This narrative review presents a picture that neurologists and the multidisciplinary team of healthcare providers can prescribe exercise as part of the comprehensive care of people with MS. We base our argument on the authors’ cumulative literature archives, which included the references list of a critical historic text *Multiple Sclerosis: The History of a Disease* [27] by Jock Murray (2004). Additionally, we searched PubMed and Scopus for relevant review articles published in English from 1st of January 1969 through 1 October 2021, and references and relevant articles. The search terms were; Article Title; “Multiple sclerosis” and “review” or “analysis”, and Article title, Abstract, Keywords; “physical activity”, “exercise” and “exercise training”. There were no language restrictions in the search criteria; however, we only reviewed published or translated papers in English.

We begin with a historical overview of the inclusion of exercise in the care of MS by neurologists. Indeed, we offer information on pivotal moments in the long-term and modern history of exercise in the care of MS patients. To that end, we separate this paper into sections, including (a) What we know about exercise in MS, including a history of benefits and safety, and (b) What we recommend neurologists and MS healthcare providers prescribe for exercise based on recent guidelines and theory, and conclude with direct actionable steps for the promotion of exercise. Our personal view is that tracing the history and potential of exercise in the clinical care of MS might open a new era of exercise promotion through the comprehensive care team, particularly the neurologist.

## 2. What We Know about Exercise in MS, Including a History of Benefits and Safety

### 2.1. Early Cases of Exercise in MS

The application of exercise among patients with MS has its roots in early disease cases, as presented in Jock Murray’s excellent text on Multiple Sclerosis: The History of a Disease [27]. For example, as indicated in a case report by pioneering Scottish neuropathologist John Abercrombie in 1838 [28];

“A gentleman aged 34, of a slender make and very active habits, was affected in the summer of 1815 with numbness and diminished sensibility of all extremities…along with diminution of muscular power. He could walk a considerable distance, though he did so with a feeling of insecurity and unsteadiness; but he could not …perform running leaping or very quick walking. He was in other respects in good health. Various remedies were employed, without benefit. He became determined to try the effect of violent exercise. For this purpose, he walked as hard as he was able, 5 or 6 miles in a warm evening, and returned home fatigued. Next morning he had severe pains in the calves of his legs, but his other complaints were much diminished, and in a few days disappeared. He has ever since enjoyed very good health”.

That case report illustrates that exercise may have long-term benefits for people with MS, but it further demonstrates the importance of careful promotion of exercise training. Our modern knowledge does suggest caution in interpretation as, for example, the implication that one session of vigorous exercise may result in complete symptom remission is misleading. Today, clinicians might suggest that the patient start more gradually and exercise in the morning during cooler temperatures rather than as suggested in the case report.

Other case reports from physicians describing the inclusion of exercise in the treatment of MS, occurred soon after the differentiation of MS from other neurological conditions, by the French School of neurology and Jean-Martin Charcot in 1868 [29]. For example, William Alexander Hammond, one of the founding members of the American Neurological Association and former Surgeon General for the United States, reported on common treatments used by physicians to treat MS in the mid-19th century, these included the use of exercise [30];

“(Hammond employed) the same therapies as others used. His approach was to give repeated courses of chloride of barium, iron, hyoscyamus, strychnine, nitrate of silver, and cod liver oil, in addition to recommending two glasses of wine daily and moderate exercise.”

Charcot, who first presented lectures on MS at the Hôpital La Salpêtrière medical asylum in Paris, was among the 19th century clinicians who included physical treatments for MS. These clinicians indicated that a clear stance on the potential value or harm of exercise for people with MS did not exist in the 19th century [27].

“Patients with neurological disease were often treated with water therapy, spas, baths and douching. Commonly used physical measures for MS were galvanic and faradic stimulation, (physical treatments were) used throughout the 19th century.”

There was no universal support for exercise training in MS, which is partly associated with the description of Uhthoff’s syndrome. Indeed, towards the end of the 19th century, German neurologist Wilhelm Uhthoff collected data from 100 clinical cases of MS. Uhthoff specialised in the eye abnormalities in MS and described a transient blurring of vision, coined exercise-induced amblyopia, in 4 of the 100 patients with MS [31]. This observation raised long-standing concern over the application of exercise in MS; it was considered that exercise-induced thermogenesis might be harmful to the disease and its progression.

In 1921, the Association for Research in Nervous and Mental Disease met in New York and discussed the current knowledge and research on MS. The delegates included leaders in neurology, particularly American neurology, with discussions based on clinical findings and written papers and textbooks on neurology. Debate on the disease course, complications, prognosis and treatment following presentation concluded in advice such as avoiding physical exertion, pregnancy and temperature extremes [32]; however, Drs. Sachs and Friedman, clinical neurologists from the Neurological Service of Mount Sinai Hospital, New York, listed helpful approaches applied in clinical practice. Early 20th-century clinical practice included warm baths, moderate and skillful massage and methodical exercise [27]. By the mid-1950s Scottish neurologist, Douglas McAlpine recommended rest and rehabilitation in a sanitorium for MS patients (22). McAlpine also suggested movement to delay a patient becoming bed-bound and exercise application to prevent ataxia and deformities [33]. Whilst exercise was being applied in clinical practice, the authoritative writings and meetings of the early 20th century implied that exercise should be avoided for people with MS [32].

The 19th and 20th-century clinical practice and writings provide a glimpse into the complicated and conflicting picture of exercise promotion in disease management and care. Some considered exercise training beneficial and essential in MS, whereas others considered exercise controversial and potentially harmful. A complicated picture of exercise promotion in MS care still exists in modern times, but that is slowly eroding through the increasing number of clinical trials supporting the benefits of exercise in MS.

### 2.2. Dawn of the Randomised Controlled Exercise Trials

The direction of MS care and research was strongly influenced by the formation of the National MS Society (NMSS), initiated by American Sylvia Lawry in 1946. Now, 138 countries have an advocacy organisation for MS [2]. There was soon a need for evidence-based treatment in MS, including exercise training. The focus on exercise in clinical trials of MS began in 1986 when Schapiro, Petajan and colleagues, neurologists at the Universities of Minnesota and Utah, respectively, designed one of the first pilot studies of exercise training in MS [34]. Schapiro’s team safely took 50 people with MS through a fitness testing protocol, and the participants underwent an incremental submaximal exercise on a cycle ergometer. Participants were randomised into either a control group that continued to live as normal or an exercise group that received educational lectures on exercise, nutrition and stress management as well as prescriptions for unsupervised, home-based moderate-intensity aerobic exercise training for 16 weeks. Overall, the study demonstrated that people with MS could engage in a regular exercise programme without adverse effects. The study was successful, and differences between pre- and post-intervention established a 10% increase in aerobic fitness for those in the exercise group. These results were more remarkable in participants with a lower disability profile (i.e., Expanded disability Status Scale ≤3.5). This finding may have set a precedent for MS exercise research’s initial bias toward persons with less disability.

The work undertaken in Utah was followed by a seminal trial funded by the NMSS and conducted by Petajan et al. [35]. Forty-six persons with MS were randomised into supervised exercise training or the wait-list control condition. The exercise stimulus was delivered three times per week for 15 weeks to 21 participants during the main study. This included 30 min of training at 60% of VO_2max_. Importantly, adherence in the exercise group was high (97% of the exercise sessions were completed), with few adverse events. Participants in the exercise group demonstrated greater improvements in fitness, muscular strength, skinfold thickness, triglyceride levels and quality of life than the control condition.

### 2.3. Growth of Clinical Trials and Expanding Knowledge of Benefits

The volume and quality of research on exercise interventions among people with MS has improved considerably, and there is now more evidence for the benefits of exercise in MS than any other neurological condition [36]; this may be due to the strong evidence for the safety of exercise in MS [37]. For persons with MS, exercise can improve physical fitness [38,39,40], walking mobility [41,42,43], strength [43,44], balance [45,46], cognition [47], fatigue [40,48,49,50,51,52,53], depressive symptoms [54,55,56] and quality of life [57,58]. There is evidence indicating that exercise has a positive effect on the hippocampus [59,60], sleep quality [61,62] and cardiovascular and metabolic comorbidity [63,64,65]. Exercise has been associated with a reduced relapse rate [66,67] and can slow disability progression [68]. The evidence for exercise training in MS is acknowledged in prestigious medical journals, including *Nature Reviews Neurology* [69] and *The Lancet Neurology* [70]. However, we note that such systematic reviews are limited by the quality of the primary studies, and the current research is hindered by small sample sizes, poor methodology and inconsistencies in reporting safety, adverse events and adherence/compliance [71].

### 2.4. Exercise Safety

The safety profile of exercise in people with MS is similar to that of the general population [66]. We know this following a systematic review by Pilutti et al. [66], who gathered data from 26 randomised controlled trials of exercise training in MS. This represented 1295 participants who engaged in exercise or control conditions. The estimated relative risk of relapse and adverse events across studies for the exercise versus control conditions were calculated as the ratio of the two rates between conditions. Overall, the relapse rates across studies were 4.6% for those in the exercise conditions and 6.3% for those in the control conditions. This corresponds to an approximately 27% lower relapse rate for exercise training versus non-exercise control conditions. Notably, the rate of other adverse events across studies was higher in the exercise conditions (2.0%) versus the control conditions (1.2%), and this represents a 67% higher risk of adverse event from exercise. Relapse has occurred in exercise studies, but in no higher a rate in those exercising than not exercising, and adverse events including illness, and joint pain, again, common in non-exercisers. The review authors point out that the risk of adverse events from exercise in MS is similar to in the general population [66]. As we have noted, the results of systematic reviews are based upon the reporting in the primary research studies, and Pilutti et al. [66] indicate that many exercise-based randomised controlled trials are in small samples. Further, many had not reported on the safety profile of the study. Hence, we take caution when considering the results from this one review, highlighting the need for further clarity of the safety of exercise in MS [71]. Exercise appears to be safe in MS, and it is associated with a slight decrease in the risk of relapse but a slight increase in the risk of adverse events.

## 3. What We Recommend Neurologists and MS Healthcare Providers Prescribe for Exercise Based on Recent Guidelines

### 3.1. Current Recommendation Guidelines

In 2013, North American researchers met to discuss the quality and results of the current exercise and physical activity clinical trials in MS for adults aged 18–64 years [57,72]. These meetings resulted in a literature review that formed the basis of the one set of guidelines for exercise in people with mild to moderate MS. These guidelines were reviewed by 500 members of the MS community, adding consumer approval, and recommend two sessions of 30 min of moderate-intensity aerobic exercise and two sessions of whole-body resistance exercise per week.

Those initial guidelines were recently updated by an international group of researchers and clinicians commissioned for creating recommendations across the disability spectrum [24,73]. These groups essentially adopted the previous guidelines and expanded them for broader use [73]. In summary, current exercise guidelines for people experiencing mild to moderate MS (EDSS ≤ 6.5) were increased to add more aerobic exercise for those already physically active and to include daily flexibility and 3–6 days of neuromotor exercise to target stability and falls prevention [24]. The recommendations are provided in Table 1. For those with severe MS disability, a summary of these recommendations includes breathing exercise every second day, daily flexibility exercise, aerobic exercise and functional strength exercise (upper and/or lower extremities) every second day and core activation exercises twice daily [24].

The recommendations [24] clearly indicated that healthcare providers should endorse and promote the benefits and safety of exercise. For those with more severe disability or for whom exercise is especially challenging, referrals to specialist physiotherapy, occupational therapy or exercise physiology are required. Traditionally, these allied health professions value therapeutic exercise [22,74], have the expertise to deliver exercise training [75] and are strategically placed to facilitate discussions around exercise training. However, evidence indicates that patients with MS have reduced access to professions such as physiotherapy [16,19,21]. If, as we suggest, prescription of exercise training begins with the neurologist and MS nurses, this will require change across all levels of the socio-ecological model of healthcare [19,21,76] to ensure equity in the promotion of exercise, and not just to patients who have access to allied healthcare therapists. National MS societies and advocacy organisations are striving to meet the exercise educational need of neurologists and multidisciplinary MS healthcare teams. Research is underway to address the learning needs of healthcare providers concerning exercise training [15,76]. One critical finding from the literature is the beneficial combination of exercise prescription alongside education and discussion on exercise behaviour change [77,78]; therefore, healthcare professionals specialising in behaviour change (e.g., occupational therapists and psychologists) may have a role in either direct patient promotion of exercise, or in education of other healthcare providers to educate them in the use of behaviour change techniques.

**Figure 1:**
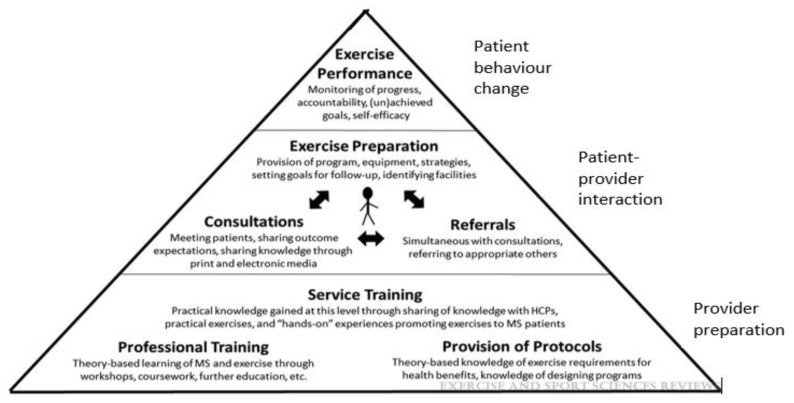
Exercise promotion conceptual model in MS care [76], reproduced by kind permission by Exercise and Sports Science Review© 2018.

For example, following qualitative research with patients and healthcare providers, including neurologists, a model of care has been proposed [76] and refined following neurologist feedback [23]. As displayed in Figure 1, the root of exercise promotion is the patient-provider interaction. To maximise this opportunity, healthcare providers require professional training and access to exercise protocols (e.g., exercise guidelines and assessments to monitor exercise performance), inter-professional consultations and referrals between the healthcare team, further inter-professional service training may be required to ensure consistency in exercise promotion.

Neurological clinicians, including physiotherapists, occupational therapists and exercise physiologists, are considered the recommended specialists in the evaluation and prescription of exercise and should be involved in the early patient evaluation to establish individualised plans for clients with MS [24]. Ongoing monitoring across neurologists, specialists nurses and these allied health exercise specialist clinicians is essential in exercise promotion. Adjustment in exercise prescription should be treated similarly to adjustment in pharmacological prescription, with an annual review involving a multidisciplinary healthcare team utilised to optimise exercise-associated positive outcomes.

To prepare neurologists and the multidisciplinary team to effectively promote exercise in people with MS there is a growing awareness of the need to put provisions in place. These include exercise guidelines based on the increasing evidence on benefits and opportunities for exercise promotion in comprehensive MS care and research to confirm exercise safety in MS.

### 3.2. Understanding Theory to Overcome Exercise Barriers

Anticipation of the client’s concerns and knowledge of potential exercise barriers will allow neurologists and healthcare providers to optimally prepare for the patient-provider interaction in relation to exercise promotion. The early age of MS onset and the whole life expectancy of persons with MS foretell the importance that timing matters and highlights the early and ongoing promotion of exercise in MS management [11,79]. Theory-based behaviour change exercise intervention represents a salient combative approach that yields optimal physical activity and exercises participation [77,80]. The use of behaviour change techniques in exercise promotion at the time of diagnosis and beyond allow neurologists and healthcare providers to help those with the greatest potential achieve lifelong benefits. Inherent in these techniques is understanding and assisting in overcoming concerns and barriers towards exercise participation.

Common barriers to exercise at the individual level include MS-related impairment and disability, attitude and motivations, knowledge of exercise benefits and environmental factors of finance, support and accessibility [81,82]. While at the healthcare organisational level, the availability of exercise-knowledgeable staff information and equipment are known barriers [19,81,82]. As such, healthcare providers and healthcare systems that (1) Become knowledgeable in the delivery of appropriate exercise to address impairments and disabilities associated with MS, (2) Utilise education and behavioural interventions to address client knowledge and exercise motivation and (3) Adopt simple strategies to deliver accessible exercise will be best placed to overcome clients concerns and potential exercise barriers [24,81].

## 4. Recommendations

We provide a clear message of the importance of exercise promotion in the care of people with MS, mainly through the neurologist and the multidisciplinary neurology healthcare team. Table 2 indicates actionable steps and suggested resources neurologists and the multidisciplinary should consider if they want to promote exercise in their practice. Table 2 table further indicates where acknowledgement of the promotion of exercise must be assisted by the wider MS community, such as persons with MS (i.e., active engagement in initial and ongoing consultation with neurologists and the multidisciplinary healthcare team related to exercise behaviours), carers (i.e., where necessary as advocates for persons with MS), healthcare systems (i.e., for referral pathways and resource development) and MS advocacy groups (i.e., for resource development and community promotion). We further provide the most recent exercise guidelines for prescription in MS care.

## 5. Conclusions

Exercise prescription has varied in the historical management of MS; however, research over the past 30 years has underscored a vital role of exercise in symptom management and potential disease modification. Patients with MS need and seek consistent messages regarding exercise training, and the neurologist is critical in this process alongside the multidisciplinary team. Exercise training is a safe intervention with no known long-term side effects. There is good adherence with exercise training, and it is beneficial for numerous symptoms and deficits in MS. Exercise training should be prescribed early and often among those with MS. Neurologists alongside the multidisciplinary team are perhaps the missing link in changing the problem of overwhelmingly low levels of participation in MS.

## Figures and Tables

**Table 1:** The current exercise guidelines for people with mild to moderate MS, reproduced by kind permission from the Canadian MS Society. © 2013. Canadian Society for Exercise Physiology. * modifications courtesy of Kim et al., 2019 [73].

	General Aerobic Activity	Advanced Aerobic Activity *	Strength Training
How often?	2/3 times per week	5 times per week	2/3 times per week
How much?	Gradually increase your activity towards being able to complete 30 min of aerobic activity during each workout session	Increase your activity towards being able to complete 40 min of aerobic activity during each workout session	Repetitions are the number of times you lift and lower a weight Try to do 10–15 repetitions of each exercise. This counts as 1 set Gradually work up to doing 2 sets of 10–15 repetitions of each exercise
How hard?	The activities should be performed at a moderate intensity. Moderate intensity physical activity is usually between 11 and 13 on the 20-point RPE scale, and it causes your heart rate to go up. Another rule of thumb, if you are doing moderate-intensity activity you can talk, but not sing a song, during the activity An alternative way of measuring moderate intensity of exercise is 40–60% VO_2peak_ or HR_peak_	The activities should be performed at a moderate to vigorous intensity. Intensity of exercise can approach 15 on the 20-point RPE scale. Another rule of thumb, if you are doing vigorous-intensity activity you can only say a few words while doing your activity. An alternative way of measuring moderate intensity of exercise is 70–80% VO_2peak_ or HR_peak_	Choose a resistance (using free weights, cable pulleys, bands etc) heavy enough that you can barely, but safely, finish 10–15 repetitions of each exercise You should rest for 1–2 min between each set and exercise, or alternate with a different resistance exercise (e.g., upper body then lower body)
How to?	Options should be feasible and sustainable, and might include		
	Some options for general aerobic activities include Upper body exercise: arm cycling, seated shadow boxing Lower body exercise: walking, leg cycling Combined upper and lower body exercises: elliptical trainer	Some options for advanced aerobic activities include Same as the general guidelines Running Road cycling	Strength training activities Weight machines Free weights Cable pulleys
	Other types of exercise that may bring benefits Elastic resistance bands Aquatic exercise Calisthetics		
Special considerations	Progressions should start with either duration or frequency and finally progress intensity per tolerability of the person Rest your muscles 2 to 4 min in between sets and muscle groups. Rest your muscles for at least 1 day between strength training sessions Aerobic and resistance training can be performed on the same day as aerobic exercise training, depending on tolerability MS Specific symptoms (i.e., fatigue and heat sensitivity) should be identified and discussed before prescribing exercise routine.		

**Table 2:** Actions and proposed resources to support healthcare providers wanting to promote exercise.

Actions and Resource Development	Target Audience
Service Training (i.e., sharing of knowledge between exercise- specialist healthcare providers with non-exercise specialist healthcare providers or behaviour change specialist healthcare providers with non-behaviour change specialist providers) to deliver evidenced guideline-based exercise promotion to patients/clients, ideally for use at initial consultations and review consultations thereafter	Neurologists and the multidisciplinary healthcare team, and healthcare team systems
Professional Training (i.e., theory based education) to deliver evidenced guideline-based exercise promotion to patients/clients, ideally undertaken at graduate level and post-graduate continual education	Neurologists and the multidisciplinary healthcare team, educational establishments and national MS advocacy organisations
Evidenced guideline-based resources or protocols to apply exercise health benefits and knowledge to design, or refer for design (by appropriate exercise specialist healthcare provider), patient/client specific programmes	Neurologists and the multidisciplinary healthcare team
Initial and ongoing consultations where exercise outcome expectations and goal discussion are salient and where knowledge can be shared in client-accessible print and electronic materials (with consideration of health-literacy and language capacity)	Neurologists and the multidisciplinary healthcare team, persons with MS, carers.
Cross-referral pathways between exercise-specialist healthcare providers/behaviour change specialist healthcare providers with non-exercise/non-behaviour change specialist healthcare providers should be developed to ensure appropriate and comprehensive content and acknowledgement ofMS symptoms (e.g., fatigue, mobility-disability, depression and cognitive impairment), personal circumstances and community circumstances which may prevent engagement in exercise.	MS healthcare providers Neurologists and the multidisciplinary healthcare team systems and national MS advocacy organisations
Exercise and behaviour change preparation through appropriate provision of exercise programme explanations, equipment and faciliries, and use of evidenced behavioral strategies, goal discussion, sequestering of barriers and mastery of life-long exercise behaviours	MS Exercise specialist providers and MS Behavioural specialist providers
Monitoring of exercise behaviour performance ideally at initial consultation and review consultations thereafter. Inclusive of monitoring functional progress, client accountability toward exercise behaviour, exercise behavioural (un) achievement and goal discussion updates.	Neurologists and the multidisciplinary healthcare team
